# Genome-Wide Identification and Expression Analysis of Polygalacturonase Gene Family in Kiwifruit (*Actinidia chinensis*) during Fruit Softening

**DOI:** 10.3390/plants9030327

**Published:** 2020-03-04

**Authors:** Wenjun Huang, Meiyan Chen, Tingting Zhao, Fei Han, Qi Zhang, Xiaoli Liu, Changying Jiang, Caihong Zhong

**Affiliations:** 1 Key Laboratory of Plant Germplasm Enhancement and Specialty Agriculture, Wuhan Botanical Garden, Chinese Academy of Sciences, Wuhan 430074, China; wjhuang@wbgcas.cn (W.H.); chenmeiyan_57@126.com (M.C.); polly828830@126.com (T.Z.); hanfei-0904@163.com (F.H.); farmzhangq@163.com (Q.Z.); jiangchangying2020@163.com (C.J.); 2 Innovative Academy of Seed Design, Chinese Academy of Sciences, Beijing 100101, China; 3 Engineering Laboratory for Kiwifruit Industrial Technology, Chinese Academy of Sciences, Wuhan 430074, China

**Keywords:** kiwifruit, *Actinidia*, polygalacturonase, pectin, cell wall, softening

## Abstract

Polygalacturonase (PG) is an essential hydrolytic enzyme responsible for pectin degradation and thus plays an important role in fruit softening and other cell separation processes. PG protein is encoded by a multigene family, however, the members of PG gene family in kiwifruit (*Actinidia chinensis*) have not been extensively identified. In this study, a total of 51 *AcPG* genes in kiwifruit genome were identified. They are phylogenetically clustered into seven clades, and of them *AcPG4* and *AcPG18* with other known *PG* genes involved in fruit softening from peach, pear, papaya and melon form a small cluster together. The members of kiwifruit *PG* gene family consist of three to nine exons and two to eight introns, and their exon/intron structures are generally conserved in all clades except the clade D and E. During fruit softening of kiwifruit ‘Donghong’ under ambient temperature, cell wall modifying enzymes, including PG, PL (pectate and pectin lyases), and PE (pectinesterase, also known as pectin methylesterase, PME) showed a different activity profile, and of them, PG and PE activities largely correlated with the change of pectin content and firmness. Moreover, only 11 *AcPG* genes were highly or moderately expressed in softening fruit, and of which three *AcPG* genes (*AcPG4*, *AcPG18,* and *AcPG8*, especially the former two) has been found to strongly correlate with the profile of PG activity and pectin content, as well as fruit firmness, suggesting that they maybe play an important role in fruit softening. Thus, our findings not only benefit the functional characterization of kiwifruit *PG* genes, but also provide a subset of potential *PG* candidate genes for further genetic manipulation.

## 1. Introduction

Polygalacturonase (PG) (EC 3.2.1.15) is an important pectin-digesting enzyme that hydrolyzes the α-1,4 bonds between adjacent galacturonic acids within the homogalacturonan backbone of pectin that constitutes the plant cell wall [1]. Pectins play a central role in the control of cellular adhesion and thereby of the rheological properties of the cell wall [2]. Therefore, PG activity has been shown to be associated with a wide range of plant developmental processes, such as fruit ripening and senescence, cell elongation, organ abscission, pod and anther dehiscence, pollen grain maturation, and pollen tube growth [1,3]. However, more attention is paid to the roles of PG in ripening fruit, particularly tomato [1,4]. For example, a number of studies aiming to retard fruit softening with the transgenic approaches to manipulate the activities of different classes of cell wall enzymes including PG have been reported with a range of success [5].

Kiwifruit, largely regarded as a climacteric fruit, behaves significantly differently from a typical climacteric fruit, such as tomato or banana [6]. The ethylene production in kiwifruit occurs at the end of the ripening process and the softening of fruit to eating firmness occurs largely ahead of the ethylene production [7]. The previous studies indicated that the pattern of kiwifruit softening is sigmoidal with three distinct phases: Phase 1 slow, Phase 2 fast, and Phase 3 slow [6,8]. Accompanied with the kiwifruit softening progress, the change of cell wall composition and the activities of cell wall enzymes occurred [8,9]. The main biochemical changes include pectin solubilisation, galactose loss, soluble pectin degradation, and a reduction in the molecular weight of xyloglucan [9]. The change of PG activity during ‘Hayward’ softening have been described previously [10,11,12], but their results seemed somewhat contradictory. The increase of PG activity in the late stage of ‘Hayward’ ripening was reported [11,12], whereas Wegrzyn and MacRae [10] reported a slight decrease. It has been extensively reported that in other fruits, such as tomato [13], peach [14], apple [15] and strawberry [16], the PG activity coincides with the softening process and become abundant during ripening.

Considering the important role of PG activity in pectin degradation and thereby cell wall disassembly and fruit softening, the study of the identification of *PG* genes in kiwifruit have been undertaken since the first report of the genomic DNA clone of *PG* gene isolated from ‘Hayward’ in 1993 [17]. To date, three cDNA clones of *PG* genes *CkPGA*, *CkPGB* and *CkPGC* (also known as *AC-PG* later) have been isolated from *A. chinensis* var. *chinensis* fruit and they showed a different expression pattern in various tissues and different stages of fruit development, and among them *CkPGC* strongly responded to exogenous ethylene treatment [18,19]. Moreover, *CkPGC* (also recently referred to as *AcPG*) has been shown to be largely up-regulated in either ethylene or low temperature-induced fruit softening [20,21]. Recently, a pair of *PG* cDNA clones from *A. eriantha* fruit *PGC1* and *PGC2* has been isolated and the former is possible to be the homologue of *CkPGC* gene [22]. Although both *AePGC1* and *AePGC2* were more highly expressed in the GP genotype than the PP genotype of *A. eriantha* during detachment development, but the PG activity was not detected in the GP genotype [22]. Thus, the function of *PG* genes in pectin degradation and thereby fruit softening of kiwifruit should be further studied for confirmation.

Plant *PG* genes belong to the large Glycoside Hydrolase Family 28 (GH28), a member of the Glycoside Hydrolase (GH) superfamily in organisms [23,24]. *PG* gene family has been identified in genomes of several plant species, such as *Arabidopsis* [23,25], *Populus* [26], apple [27] and peach [28]. Their results suggested that the whole genome and segmental duplications contribute to the expansion and functional diversification of *PG* gene family members. However, it is clear that only a part rather than all of PG family members in kiwifruit genome have been currently identified, and their functional characterizations, especially the role in fruit softening have still been relatively limited. The manually annotated high-quality genome database of kiwifruit (Red5_PS1_1.69.0) has been recently released [29], which provides a more reliable genome information and makes the genome-wide identification of *PG* gene family in kiwifruit available accurately. Therefore, in this study, 51 members of *PG* gene family from this recently released kiwifruit Red5 genome were successfully identified and their expression patterns during kiwifruit ‘Donghong’ softening process were also investigated to correlate themselves with the PG enzyme activity and the pectin content. Finally, three *AcPG* genes (*AcPG4*, *AcPG8* and *AcPG18*) have been identified to be possibly involved in pectin degradation and kiwifruit softening.

## 2. Results

### 2.1. Genome-Wide Identification of Polygalacturonase (PG) Family Members in Kiwifruit

**Figure 1:**
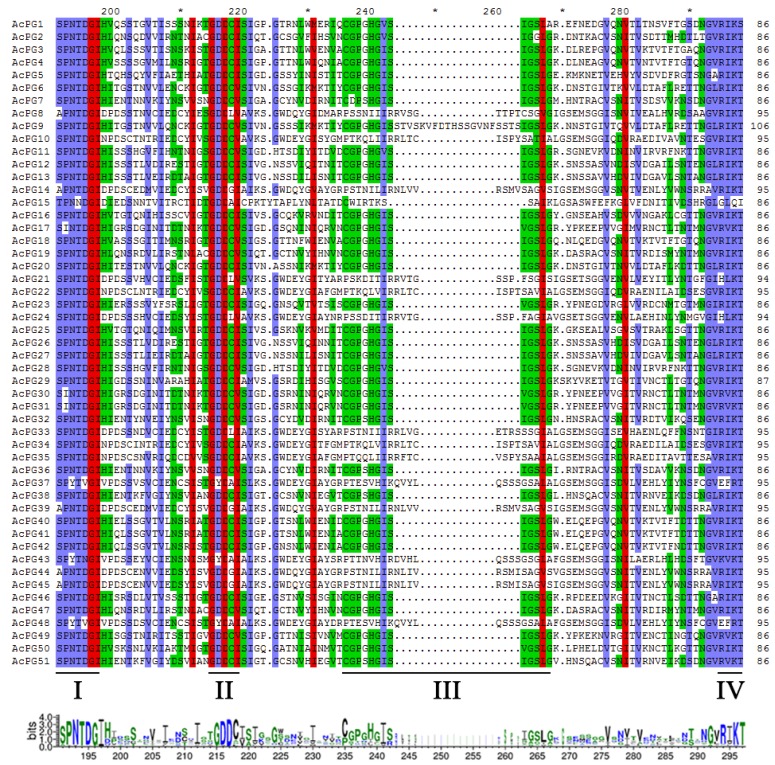
Multiple sequence alignment of peptides of kiwifruit PGs containing the four conserved domains. The underlines indicate the four typical conserved domains of PGs, referred to as domain I, II, III and IV as previously reported (Qian et al., 2016). Different shading colors illustrate different similarities (red: 100%, blue: ≥80%, green: ≥60%). The consensus sequence is shown at the bottom by letter logos. The bit score indicates the relative frequency of each amino acid at that position.

Two blast methods were used to identify all potential *PG* genes in kiwifruit genome. Firstly, a BLASTP search against kiwifruit Red5 genome database using 66 PG protein sequences from *Arabidopsis* genome and 4 known PG protein sequences from kiwifruit yielded 53 PG candidates. Secondly, the GH28 HHM profile search against kiwifruit Red5 genome database produced 52 PG candidates. These two search approaches generated a total of 53 PG candidates together, and one candidate (CEY00_Acc03902.1) was removed out due to the absence of GH28 domain via CDD and Pfam database search. Subsequently, another candidate (CEY00_Acc15270.1) was further excluded due to the lack of at least any two domains of the four highly conserved PG domains (I to VI) of PG proteins. Finally, a total of 51 PG family members were identified in kiwifruit genome and named *AcPG1* to *AcPG51* according to their chromosomal locations (Table 1). The peptides of 51 AcPG family members spanning the four highly conserved domains of PG proteins were aligned, and the results showed that 34 members contained the conserved domains I, II, III, and IV, while 17 members lacked the domain III, and of which only AcPG15 further lacked the domain IV (Figure 1). The sequence analysis results indicated that the open reading frame (ORF) lengths of the 51 kiwifruit PG family members ranged from 1029 to 1599 bp, and their deduced peptide sequences varied in length from 342 to 532 amino acids with a predicted isoelectric point (pI) varying from 4.69 to 9.59 and a molecular weight (Mw) varying from 36.60 kD to 57.85 kD (Table 1). Moreover, the four known PG proteins isolated previously from kiwifruit including AdPG, CkPGC, AePGC1 and AePGC2 were also blasted against protein sequences of 51 AcPGs and their top hits with highest identities were AcPG16 (95%), AcPG18 (99%), AcPG18 (97%), and AcPG4 (97%), respectively.

### 2.2. Phylogenetic Analysis of PG Family Members in Kiwifruit

**Figure 2:**
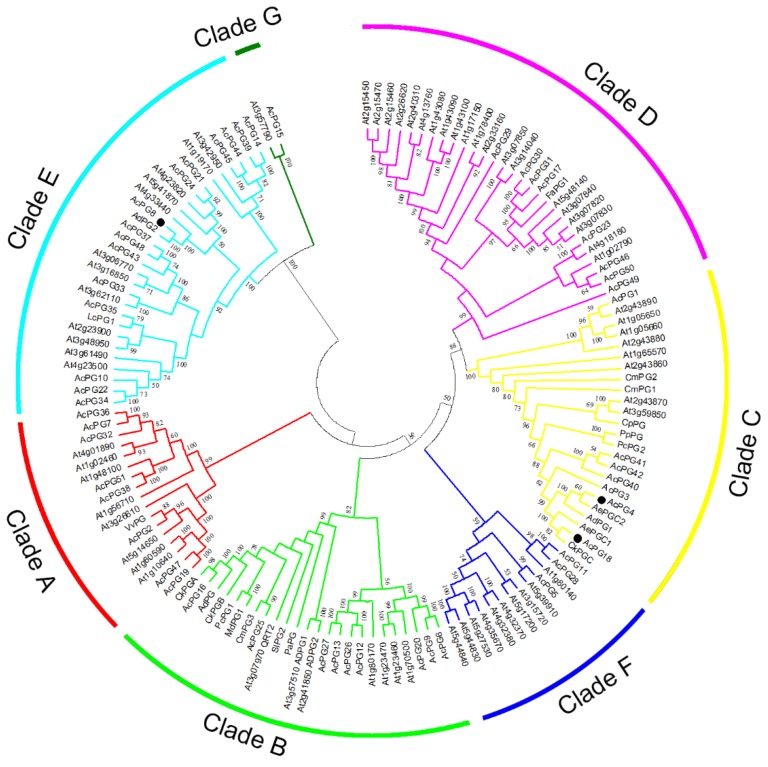
Phylogenetic relationship analysis of PGs from kiwifruit and other plants. Multiple alignments of full-length protein sequences of PGs from kiwifruit and other plant species were performed by Clustal W, and the phylogenetic tree was constructed using MEGA 6.0 by the Neighbor-Joining method. Numbers on branches indicate the bootstrap percentage values from 1000 replicates, and values lower than 50 are hidden in the phylogenetic tree. Three PG genes from kiwifruit (*AcPG4*, *AcPG8* and *AcPG18*) were indicated by black circles. The protein sequences of *AtPG* gene family members from *Arabidopsis thaliana* were retrieved from TAIR database, and the protein sequences of other functionally known *PG* genes from fruits involved in fruit softening were downloaded from GenBank database and their sequence information are as follows: kiwifruit AdPG (AAC14453.1), CkPGA (AAF71160.1), CkPGB (AAF71156.1), CkPGC (AAF71158.1), AdPG1 (AYP70925.1), AdPG2 (AYP70310.1), AePGC1 (ARA90624.1) and AePGC2 (ARA90625.1), apple MdPG1 (AAA74452.1), avocado PaPG (CAA47055.1), grape VvPG (ABW76153.1), litchi LcPG1 (AFW04075.1), melon CmPG1 (AAC26510.1), CmPG2 (AAC26511.1) and CmPG3 (AAC26512.1), papaya CpPG (ACH82233.1), peach PpPG (CAA54150.1), pear PcPG1 (BAC22688.1) and PcPG2 (BAC22689.1), strawberry FaPG1 (ABE77145.1) and tomato SlPG2 (NP_001234021.1).

Fifty-one members of *PG* gene family identified from kiwifruit Red 5 genome, 66 *PG* family members from *Arabidopsis* and 21 other functionally known *PG* genes from horticultural plants possibly involved in fruit softening were used to construct the phylogenetic tree of *PG* genes. The results indicated that all *PG* genes were clustered into seven clades and designated as the Clade A to G (Figure 2), according to the previous report [28,30]. Specifically, the Clade E contained the largest members of kiwifruit PG family with 15, the Clade F and G only consisted of three and one members, respectively, and the other clades harbored seven to nine members (Figure 2). *AcPG4* and *AcPG18* showed a closer relationship with the previously isolated *PG* genes of kiwifruit *CkPGC*, *AdPG1*, *AePGC1* and *AePGC2*, as well as other known *PG* genes from peach, pear, melon and papaya, and they all were clustered into the Clade C (Figure 2). Meanwhile, most of the other known *PG* genes from tomato, apple, avocado, and pear were located in the Clade B, which also contained *AcPG16* and *AcPG25* of kiwifruit (Figure 2). However, the known *PG* genes from grape, strawberry and litchi were placed into the Clade A, D and E, and their phylogenetically close PG members from kiwifruit genome are *AcPG2*, *AcPG30/31/17*, and *AcPG35*, respectively (Figure 2). In addition, more than ten pairs of kiwifruit PG proteins revealed a high degree of homology in the terminal nodes, such as AcPG7-AcPG36, AcPG6-AcPG9, AcPG30-AcPG31, AcPG37-AcPG48 and AcPG11-AcPG28 from different clades (Figure 2), suggesting that they might be putative paralogous genes in the kiwifruit genome.

### 2.3. Genome Distribution, Gene Structure and Conserved Motif Analysis of PG Family Members in Kiwifruit

All kiwifruit *PG* genes were mapped to the Red5 reference genome and the result showed that PG family members were unevenly distributed on only 20 out of 29 chromosomes with an average of 2.55 genes per chromosome (Supplementary Figure S1). Among these chromosomes, Chr12, Chr17 and Chr 29 contained the largest numbers of PG genes with five, while only Chr5, Chr8, Chr19 and Chr28 harbored the smallest number with one. In addition, only one tandem duplication site *AcPG40*, *AcPG41* and *AcPG42* on Chr22 was found (Figure S1).

**Figure 3:**
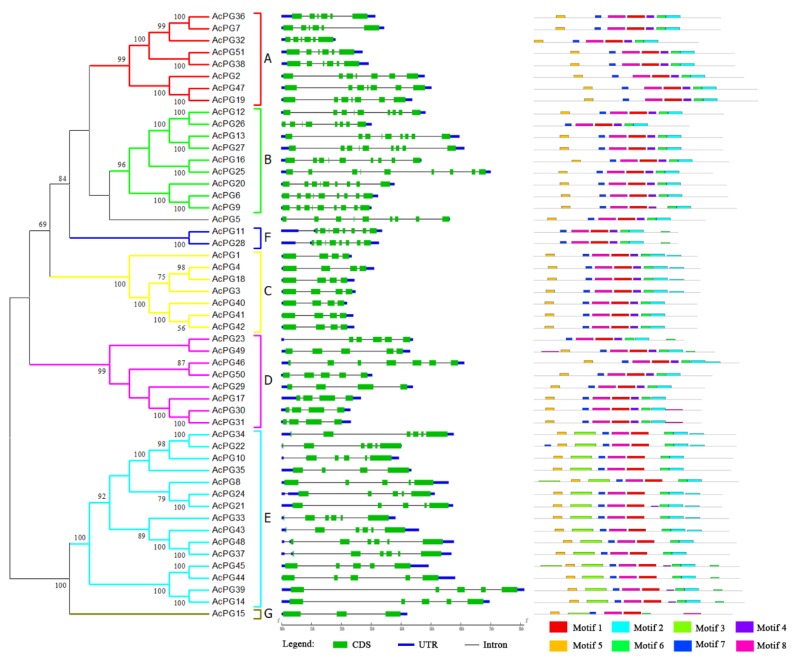
Phylogenetic tree, genomic structure and conserved motif analysis of kiwifruit *PG* genes. The left illustrates the phylogenetic tree of kiwifruit *PG* genes constructed using MEGA 6.0 by the Neighbor-Joining method with the 51 full-length protein sequences of *AcPGs*. Numbers on branches indicate bootstrap percentage values calculated from 1000 replicates, and values lower than 50 are not shown. Seven clade (A to G) are distinctly formed in the phylogenetic tree. The middle represents the exon/intron genomic structure of *AcPGs*. CDS and UTR are indicated by green and blue boxes, respectively, and intron by black line. Their sizes can be estimated using the length scale at the bottom. The right indicates the composition and position of the conserved motifs of *AcPGs*. Eight conserved motifs identified by MEME are indicated by different colored boxed. The height of each box represents the conservation of each motif.

The phylogenetic tree of *PG* genes in kiwifruit genome was constructed using the overall protein sequences and seven distinct clades were also formed (Figure 3A), which was strongly similar with that of *PG* genes from *Arabidopsis* and kiwifruit mentioned above. The exon/intron genomic structures of kiwifruit *PG* genes were also analyzed using the online tool GSDS. The results showed that *AcPG* genes consisted of three to nine exons and two to eight introns, and the genomic structure types containing four, six, and nine exons had relatively larger members with 12, 12, and 11, respectively (Figure 3B). The members of clade B and F generally contained more exons and introns. Moreover, the exon/intron genomic structures were basically conserved in each clade except for clades D and E (Figure 3B).

To further analyze conserved motifs in the amino acid sequences of kiwifruit *PG* genes, 51 kiwifruit PG protein sequences were aligned using the online tool MEME to output eight conserved motifs (Table S1). The Motif 1, 7 and 8 were found in all 51 PG protein sequences, and the Motif 2 and 6 were absent in only AcPG15 (Figure 3C). Among these motifs, the Motif 1 represented the highly conserved domain I and II of PG genes, and the Motif 6 corresponded to the domain IV. The Motif 4 corresponding to the domain III were found in 34 PG proteins. Interestingly, the Motif 3 was found to be present only in members of clade E (Figure 3C). Moreover, the composition and location order of these conserved motifs in PG protein sequences were generally similar in certain clades, especially clade C (Figure 3C).

### 2.4. Fruit Firmness, SSC, Pectin Content and PG Enzyme Activity Changes During Kiwifruit Softening

**Figure 4:**
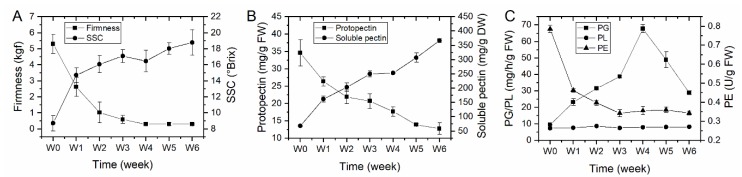
Fruit components, pectin content and activities of pectin degrading enzymes during kiwifruit ‘Donghong’ softening. (**A**) Fruit firmness and soluble solids concentration (SSC) measurement. (**B**) Protopectin (water insoluble pectin) and water soluble pectin content measurement. (**C**) Pectin degrading enzymes including PG (polygalacturonase), PL (pectate and pectin lyases) and PE (pectinesterase) activity measurement. Three biological samples were used for each analysis. Data are the means ± standard deviation (SD) bar.

In order to identify the potential role of PG enzyme in kiwifruit softening process, ‘Donghong’ fruit were placed for up to six weeks under ambient temperature to soften naturally, and firmness, SSC (soluble solids concentration), pectin content and the activities of pectin degrading enzymes including PG, PE (pectinesterase, also known as PME, pectin methylesterase) and PL (pectate and pectin lyases) were monitored every one week. The results showed that firmness declined fast to the eating firmness level (ca. 1 kgf) in first two weeks and slowed down in subsequent storage, while SSC showed a contrary change, increasing fast in first two weeks and continue to rise up slowly towards the end of storage (Figure 4A). The protopectin (water insoluble pectin) content gradually decreased and the amount of soluble pectin gradually increased throughout the entire storage of softening (Figure 4B). However, the pectin degrading enzymes showed a different expression pattern. The activity of PG enzyme firstly rose up to the peak after four weeks of storage and then declined largely, but the PE enzymatic activity decreased dramatically within the first three weeks, especially the first week and then become stable at a low level throughout the end of storage (Figure 4C). In addition, the PL enzymatic activity always stabilized at a very low level during fruit softening (Figure 4C). It is clear that the change of pectin is highly correlated with fruit firmness, and the activities of PG and PE largely coincided with the change of pectin.

### 2.5. Expression and Correlation Analysis of PG Family Members During Kiwifruit Softening

**Figure 5:**
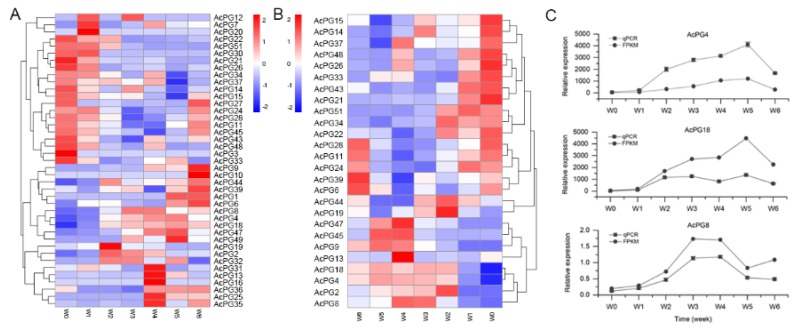
Expression pattern analysis of selected *AcPG* genes during kiwifruit ‘Donghong’ softening. Heat maps showing the hierarchical clustering of 41 *AcPGs* based on their expression patterns determined by transcriptome analysis (**A**) or the hierarchical clustering of 26 selected *AcPG* genes based on their expression patterns determined by qPCR assay (**B**), respectively. (**C**) The expression profiles of three potential *AcPG* genes probably involved in pectin degradation (*AcPG4*, *AcPG8* and *AcPG18*) are shown.

The expression patterns of 51 *AcPG* genes during ‘Donghong’ softening were also analyzed using FPKM values from transcriptome sequencing. The results showed that a total of 41 *AcPG* genes had a positive value of FPKM, and among them only 14 members (*AcPG4*, *−8*, *−11*, *−14*, *−15*, *−18*, *−21*, *−24*, *−28*, *−33*, *−39*, *−44*, *−45* and *−48*) possessed a FPKM value greater than 1, which were considered to be relatively highly expressed in fruit (Supplementary Materials Table S2). Based on their FPKM expression profiles during softening, the cluster analysis was further constructed (Figure 5A). The results showed that two larger groups were generally formed, one group members were mainly expressed at W0 or W1 early stage of softening, while the other one group members were mostly expressed at W4 or W5 or W6 late stage of softening (Figure 5A). In particular, *AcPG4*, *AcPG8*, and *AcPG18* were clustered together and their transcript levels first increased largely and then decreased, showing a generally similar expression pattern with the change of PG enzyme activity (Figure 5A and Figure 4C). In order to further confirm the results obtained from transcriptome analysis and the correlation between gene expression and enzymatic activity, a total of 26 *PG* genes containing those 14 highly expressed *PG* genes from transcriptome analysis were selected for qPCR assay and their results were shown in Figure 5B and Supplementary Table S3. Eleven out of 26 PGs (*AcPG4*, *−8*, *−11*, *−14*, *−15*, *−18*, *−21*, *−24*, *−28*, *−33*, *−39*, *−44*, *−45* and *−48*) are relatively highly or moderately expressed during fruit softening, especially *AcPG4,* and *AcPG18* with the highest expression levels (Table S3). Most of the 26 *PGs* from qPCR results showed a similar cluster with that of the 41 *PGs* from transcriptome results, and they were also mainly expressed in either early or late stage of softening. A small cluster of *AcPG4*, *AcPG8* and *AcPG18* was also formed (Figure 5B). Moreover, the correlation analysis between qPCR assay and FPKM transcriptome assay indicated that 20 out of 26 *PGs* had a good Pearson coefficient greater than 0.8, mostly greater than 0.9 (Supplementary Materials Table S4), suggesting the strong reliability of FPKM transcriptome results. Subsequently, based on the expression patterns of 26 *PGs* determined by qPCR assay and the PG enzyme activity during fruit softening, their correlation was also analyzed to identify the potential PG genes responsible for pectin degradation and thereby fruit softening. The results showed that seven *PG* genes (*AcPG4*, *−8*, *−9*, *−13*, *−18*, *−45,* and *−47*) had a good correlation, particularly *AcPG4*, *AcPG8* and *AcPG18* showing a similar expression pattern with the change of PG enzyme activity and also having a high expression level (Figure 5C and Supplementary Table S4). Meantime, these three *PG* genes were also correlated with the change of pectin and firmness, especially *AcPG4* and *AcPG18* (data not shown). Finally, these results suggested that *AcPG4*, *AcPG8,* and *AcPG18* genes are likely to be involved in pectin degradation and fruit softening.

## 3. Discussion

Plant PGs are multifunctional proteins encoded by a large gene family. Members of the *PG* gene family have been genome-widely identified in several species, such as *Arabidopsis* with 66 [23], *Populus* with 75 [26], peach with 45 [28], and soybean with even more than 100 [31]. In this study, we identified a total of 51 members of *PG* gene family utilizing the recently released and manually annotated kiwifruit Red5 genome database. As we know, the expansion and diversification of PG family is attributed to whole genome and segmental duplications. More than ten pairs of paralogous gene of PG family in kiwifruit were found, probably deriving from gene duplication (Figure 2). The phylogenetic tree of plant PGs can be divided into either three or seven clades by different scientists [23,28]. The PG family members of kiwifruit were placed into seven clades together with *Arabidopsis* PG family members and other fruit ripening-related PGs (Figure 2). The member distribution in seven clades totally agreed with the previous report that the Class I corresponding the Clade A-D and Clade F classified here had the highest members, while the Class II and III corresponding the Clade E and G, respectively, contained relatively conserved and few members in both *Arabidopsis* and *Populus* [26]. These results further confirmed the rapid expansion of PG family members occurred in the Class I. In addition, a number of known *PG* genes involved in fruit softening were supplemented into the phylogenetic tree construction in order to figure out the potential PG candidates responsible for kiwifruit softening. Majority of them were placed into the Clade B and C (Figure 2), the former harbored kiwifruit *AcPG4* and *AcPG18* and other known PGs from peach, pear and papaya, and the latter contained kiwifruit *AcPG16* and *AcPG25* and other ripening-associated PGs from tomato, apple and avocado.

Cell wall disassembly is the main factor contributing to fruit softening and textural changes [32]. It is well documented that cell wall disassembly is caused by the synergistic actions of a multitude of cell wall enzymes. In kiwifruit ‘Hayward’, the activities of cell wall associated enzymes and the change of cell wall component have been extensively studied [8,9,33]. It is mainly concluded that the activity of PG enzyme increases with ripening process and becomes abundant when fruit ripen [11,12], although a slight increase of PG activity during kiwifruit softening was previously reported [10]. Similarly, in our study, the PG enzyme activity also increased with storage time and peaked at ripe firmness, but declined and still maintained at a high level in subsequent periods (Figure 4C). Meantime, the PE activity has been shown to strongly correlate with flesh firmness, and they both decreased fast at the early stage of ripening and slowed down (Figure 4A,C). It has been suggested that the function of PE enzyme prepared unesterified galacturonic acid unit as substrates for further PG hydrolyzation [34]. A previous report indicated the PE activity increased during a very short period of ethylene treatment (Phase 1) and then drop rapidly to low levels as ‘Hayward’ fruit softened [10]. In this study, the lack of increase of PE activity in Phase I of ‘Donghong’ fruit maybe resulted from the absence of Phase I detection, probably due to the large checking period of every one week. However, the content of insoluble pectin gradually decreased and water soluble pectin (WSP) gradually increased with fruit softening (Figure 4B), which well coincided with the change of PE and PG activities, respectively. A recent study also reported a similar pattern of the pectin change during softening, which revealed an increase of WSP and a decrease of covalent binding insoluble pectin as ‘Hayward’ fruit ripened under air control, ethylene or 1-MCP treatments [33]. These results suggested that PE activity is probably responsible for pectin solubilisation, making the pectin susceptible to further degradation by PG. The similar results have been also reported from ripening tomato [35]. In addition, the PL activity were not largely changed throughout ‘Donghong’ fruit softening (Figure 4C), suggesting that it seems probably not involved in pectin breakdown, at least not playing an important role in ‘Donghong’ fruit softening. Similar to PG enzyme, PL also showed a different role in fruit softening among different species. Increasing evidence demonstrated that PG activity is not necessary or sufficient for tomato softening, but silencing a PL gene in tomato caused to reduce fruit softening and improve shelf life [1,4].

The expression profiles of kiwifruit PG family members during ‘Donghong’ fruit ripening were analyzed and their results indicated a remarkable expression divergence (Figure 5). The maximum mRNA abundance of each *AcPG* gene occurred at the different stage of ripening, and two large group were roughly formed according to their high expression levels observed at either early or late stage of ripening (Figure 5). It has been extensively reported that the enzyme activity and transcript level of PGs usually increase during ripening in several fruits, such as tomato [34], apple [36], peach [28], strawberry [16,37], and papaya [38]. Hence, it was emphasized to search the potential *PG* genes with such a similar expression pattern during ‘Donghong’ softening, although a number of *AcPG* genes showed a contrary expression profile and did not coincide with PG activity (Figure 5 and Table S4). Based on the correlation analysis between gene expression and enzyme activity of PG, seven *AcPGs* candidates were first screened out, and three out of them (*AcPG4*, *AcPG18* and *AcPG8*) were further found to well correlate with the change of pectin and firmness and also be highly expressed during softening fruit, especially *AcPG4* and *AcPG18* (Figure 5 and Supplementary Table S4). Meanwhile, it has been previously described that the *PG* genes isolated from kiwifruit ‘Hayward’, *CkPGC* (also referred as *AcPG*) and *AdPG1* were induced by ethylene or suppressed by 1-MCP, and positively associated with fruit ripening and softening [18,19,20,33]. AcPG4 and AcPG18 showed 97% and 99% identities with AdPG1 and CkPGC at amino acid level, respectively, and they all were clustered into the Clade C together with other known PGs from fruit (Figure 2). These results strongly suggest that *AcPG4* and *AcPG18* are possibly involved in pectin breakdown and fruit softening of kiwifruit ‘Donghong’. Each pair of *AcPG4*/*AdPG1* and *AcPG18*/*CkPGC* genes is possibly located at the same genetic locus, as their very tiny difference at protein sequence maybe resulted from the different genotypes or cultivars. In addition, *AcPG8* was phylogenetically close to *AdPG2,* which is functionally unclear to date, and both they were clustered into the Clade E (Figure 2). One member of the Clade E, *LcPG1* from litchi were found to play an essential role in the process of fruitlet abscission [39]. Thus, the further studies are needed to clarify the role of *AcPG8*, together with *AdPG2* in kiwifruit ripening and softening.

## 4. Materials and Methods

### 4.1. Plant Materials

Kiwifruit (*Actinidia chinensis* var. *chinensis* cv. ‘Donghong’) were harvested in September 2017 from an orchard in Pujiang county, Sichuan province, China when SSC in fruit was greater than 8 ^o^Brix, and transported under ambient temperature to the laboratory in Wuhan Botanical Garden, Chinese Academy of Sciences. The sound fruit with uniform size and shape were selected for further study.

### 4.2. Genome-Wide Identification of PG Family Members in Kiwifruit

The manually annotated kiwifruit genome database (Red5_PS1_1.69.0) was preferably used as the reference genome for exploring PG family genes due to the great improvement of gene model prediction [29]. The kiwifruit Red5 genome data (GCA_003024255.1_Red5_PS1_1.69.0) was retrieved from NCBI (https://www.ncbi.nlm.nih.gov/genome/16401?genome_assembly_id=369962). In order to identify all potential members of *PG* family in kiwifruit, two blast approaches were used. First, 66 PG proteins from *Arabidopsis thaliana* retrieved from TAIR database and four known representative PG proteins from kiwifruit (*A. chinensis* var. *chinensis*, *A. chinensis* var. *deliciosa* and *A. eriantha*) downloaded from GenBank, including AdPG (AAC14453.1), CkPGA (AAF71160.1), AePGC1 (ARA90624.1) and AePGC2 (ARA90625.1) were used as query sequences to blast kiwifruit Red5 protein database [17,18,19,22]. Second, the Glycosyl Hydrolase family 28 (GH28) Hidden Markov Model (HMM) profile (Accession No. PF00295) was retrieved from Pfam database (http://pfam.xfam.org/family/PF00295#logoBlock) and also used as query to search kiwifruit Red5 protein database. Then all the candidates of PG family members were further analyzed using Pfam and CDD database to confirm the presence of the GH28 domain. Finally, those candidates of PG family members that contained at least two highly conserved domains of the domain I, II, III, and IV of PG proteins [28,40] were regarded to be PG genes for further study.

### 4.3. Multiple Sequence Alignment, Phylogenetic Analysis, Genomic Structure and Motif Analysis

Multiple sequence alignment of PG protein sequences were analyzed using ClustalW program [41] and the alignment result was decorated by Genedoc [42] and the consensus sequence logo was generated with the online software WebLogo (http://weblogo.berkeley.edu/) [43]. The phylogenetic trees were constructed with MEGA6.0 software using the neighbor-joining (NJ) method with 1000 replicates [44]. The exon/intron genomic structures of kiwifruit PG genes were generated using the web-based bioinformatics tool GSDS 2.0 (http://gsds.cbi.pku.edu.cn/index.php) [45]. MEME motif analysis was carried out (http://meme-suite.org/tools/meme) to identify the conserved motifs of PG protein sequences [46]. Only the maximum number of motifs to determine in the MEME program was adjusted to eight. The default parameters were used for these bioinformatic tools unless otherwise specified. Genes were mapped on chromosome by identifying their chromosomal positions provided in kiwifruit genome database (Red5_PS1_1.69.0) [29]. The distribution of *AcPG* family members throughout the kiwifruit genome was drawn manually to scale.

### 4.4. Firmness, SSC, Pectin Content and Pectin Degrading Enzyme Activities Measurement

Kiwifruit ‘Donghong’ was stored for up to six weeks at ambient temperature (2 °C ± 1 °C) and 12 fruit was taken out at one week interval for measurement of firmness and SSC. Fruit firmness was measured using a GY-4 penetrometer (TOP instrument, Zhejiang, China) with a 7.9 mm probe after the removal of skin and flesh to a depth of approximately 1 mm at the equator of fruit. SSC in juice squeezed from the equator of fruit was determined using a hand-held PAL-1 refractometer (Atago, Tokyo, Japan). After SSC and firmness measurement, the flesh of each four fruit were roughly equally pooled together as a sample and frozen in liquid nitrogen and stored at −70 °C for further analysis, including pectin content and enzymatic activity measurement, and RNA extraction for transcriptome sequencing and qPCR assay. Three biological replicates for each analysis were used.

Pectin in flesh was extracted and hydrolysed into galacturonic acid and then measured using carbazole colorimetric method. Specifically, the content of pectin including protopectin (water insoluble pectin) and water soluble pectin (WSP) was determined by protopectin assay kit and WSP assay kit (Suzhou Comin Biotechnology, Suzhou, China), respectively, following the supplier’s instructions. The PG enzymatic activity was determined with 3,5-dinitrosalicylic acid (DNS) colorimetric method using PG assay kit (Suzhou Comin Biotechnology, Suzhou, China), in which polygalacturonic acid was used as a substrate to produce galacturonic acid that was then measured by DNS colorimetry at 540 nm. The amount of galacturonic acid per hour per gram fresh weight at 40 °C and pH 6.0 conditions was produced to represent the PG activity. The activity of PL enzyme was measured by spectrophotometric method using PL assay kit (Suzhou Comin Biotechnology, Suzhou, China), in which pectin from citrus peel was hydrolysed by PL sample to generate 4,5-unsaturated oligogalacturonides that was monitored for absorbance at 235 nm, and the PL activity was defined as the amount of unsaturated product per hour per gram fresh weight under 40°C and pH 5.5 conditions. Meanwhile, the activity of PE enzyme was determined by potentiometry method using PE assay kit (Suzhou Comin Biotechnology, Suzhou, China) and one unit of enzyme activity was defined as the volume (ml) of NaOH consumed to maintain pH 7.0 of reaction per sec per gram fresh weight. It was noted that the leupeptin solution was added to the extraction buffer of these enzyme assay kits to inhibit the protease actinidin according to the previous report [10]. Three biological samples for each analysis were used.

### 4.5. Transcriptome Sequencing and qPCR Assay

Fruit samples collected from kiwifruit ‘Donghong’ at seven different stages (W0 to W6) of softening were used to extract RNA. The quantity and quality of RNA were determined by a Qubit 2.0 Fluorometer (Life technologies, Carlsbad, CA, USA) and electrophoresis in 1% agarose gel. A total amount of 1 μg RNA per sample was used to construct cDNA sequencing library following the NEBNext Ultra RNA Library Prep Kit for Illumina (NEB, Ipswich, MA, USA) recommendations and they were sequenced on an Illumina HiSeq platform by Novogene Company (Beijing, China). The raw reads were processed and filtered to obtain clean reads and they are mapped to the kiwifruit reference genome database (Red5_PS1_1.69.0) [29] using HISAT2 v2.0.4 tool [47]. Gene expression level was analyzed using HTSeq v0.9.1 tool and their expression quantifications was represented by FPKM values (expected number of Fragments Per Kilobase of transcript sequence per Millions base pairs sequenced) [48]. Three biological samples for each stage were used. At last, the FPKM profiles of 51 kiwifruit *AcPG* genes during ‘Donghong’ softening were extracted for further gene expression analysis.

As for qPCR assay, RNA solution was first digested using gRNA eraser (Takara, Dalian, China) to remove any contaminated genomic DNA and then reversely transcribed by PrimeScript RT reagent kit (Takara, Dalian, China) to synthesize cDNA template following the manufacturer’s manual. The qPCR reaction was conducted with SYBR Premix Ex Taq II (Tli RNase H Plus) Kit (Takara, Dalian, China) and performed on an ABI7500 Fast Real-Time PCR equipment (Applied Biosystems, United States) following the supplier’s instruction. The qPCR program was as follows: 95 °C for 30 s, 40 cycles of 95 °C for 3 s, and 60 °C for 30 s, and a default melt curve program. Kiwifruit *AcActin* gene (CEY00_Acc08081) was selected as the reference gene. Relative expression levels of genes were analyzed using the comparative Ct method [49]. Each sample was analyzed in triplicate. Primers used for qPCR assay were listed in Table S5.

## 5. Conclusions

In summary, a total of 51 members of *PG* gene family in kiwifruit were identified through genome-wide analysis, and they were phylogenetically clustered into seven clades. During the natural softening of kiwifruit ‘Donghong’, the activities of PG and PE were generally correlated with the pectin content and firmness changes, and the expression patterns of three *PG* genes (*AcPG4*, *AcPG18* and *AcPG8*, especially the former two) has been shown to largely correlate with the profile of PG activity and thereby fruit softening. Our results indicated the genome-wide identification of *PG* gene family members in kiwifruit and the strongly potential PG genes involved in pectin breakdown and fruit softening.

## Figures and Tables

**Table 1:** Summary of PG gene family members identified in kiwifruit.

Gene Name	Gene Locus	Chromosome	Location	Length (aa)	MW(kDa)	pI	Domains	SG	Exons
*AcPG1*	CEY00_Acc01961	2	6399144–6401490	390	41.84	9.13	I, II, III, IV	C	4
*AcPG2*	CEY00_Acc02429	2	13133935–13138730	499	53.13	5.35	I, II, III, IV	A	7
*AcPG3*	CEY00_Acc03125	3	5883379–5885855	396	41.85	8.83	I, II, III, IV	C	4
*AcPG4*	CEY00_Acc03126	3	5889856–5892955	396	41.70	5.95	I, II, III, IV	C	4
*AcPG5*	CEY00_Acc05726	5	12541736–12547383	407	45.04	7.22	I, II, III, IV	F	9
*AcPG6*	CEY00_Acc06176	6	1592–4821	462	49.88	5.17	I, II, III, IV	B	9
*AcPG7*	CEY00_Acc06629	6	5704363–5707803	443	48.47	7.49	I, II, III, IV	A	6
*AcPG8*	CEY00_Acc08039	7	9542953–9548541	485	52.13	5.79	I, II, IV	E	5
*AcPG9*	CEY00_Acc08167	7	16576567–16579592	482	52.00	5.43	I, II, IV	B	9
*AcPG10*	CEY00_Acc09155	8	15928767–15932695	473	51.52	5.74	I, II, IV	E	6
*AcPG11*	CEY00_Acc12552	11	12222609–12225987	342	36.60	6.77	I, II, III, IV	F	9
*AcPG12*	CEY00_Acc12561	11	12277850–12282681	451	48.74	7.78	I, II, III, IV	B	9
*AcPG13*	CEY00_Acc12562	11	12285317–12291285	449	49.15	8.92	I, II, III, IV	B	9
*AcPG14*	CEY00_Acc12941	12	401281–408240	499	55.94	8.44	I, II, IV	E	5
*AcPG15*	CEY00_Acc13137	12	3045053–3049259	472	52.31	6.20	I, II	G	3
*AcPG16*	CEY00_Acc13161	12	3406433–3411128	465	50.49	5.07	I, II, III, IV	B	9
*AcPG17*	CEY00_Acc13599	12	12593335–12595992	399	42.65	8.70	I, II, III, IV	D	4
*AcPG18*	CEY00_Acc13940	12	18014662–18017102	396	41.67	8.83	I, II, III, IV	C	4
*AcPG19*	CEY00_Acc14204	13	2561040–2565419	532	57.85	8.22	I, II, III, IV	A	7
*AcPG20*	CEY00_Acc14205	13	2574233–2578029	459	49.65	5.15	I, II, III, IV	B	9
*AcPG21*	CEY00_Acc15593	14	3403644–3409382	447	49.18	8.92	I, II, IV	E	5
*AcPG22*	CEY00_Acc15651	14	4025837–4029852	480	52.47	4.75	I, II, IV	E	6
*AcPG23*	CEY00_Acc16366	14	17787820–17792226	357	38.23	9.03	I, II, III, IV	D	6
*AcPG24*	CEY00_Acc16389	15	268316–273441	447	48.53	6.31	I, II, IV	E	6
*AcPG25*	CEY00_Acc17075	15	10144996–10152010	426	45.99	9.04	I, II, III, IV	B	9
*AcPG26*	CEY00_Acc17817	16	2768646–2771660	370	40.11	6.73	I, II, III, IV	B	8
*AcPG27*	CEY00_Acc17818	16	2774118–2780250	449	49.13	8.91	I, II, III, IV	B	9
*AcPG28*	CEY00_Acc17848	16	3051196–3054461	342	36.73	6.29	I, II, III, IV	F	9
*AcPG29*	CEY00_Acc19095	17	3704038–3708434	407	43.82	8.85	I, II, III, IV	D	4
*AcPG30*	CEY00_Acc19265	17	7674742–7677060	399	42.56	6.06	I, II, III, IV	D	4
*AcPG31*	CEY00_Acc19279	17	7994174–7996509	399	42.54	6.06	I, II, III, IV	D	4
*AcPG32*	CEY00_Acc19706	17	10320942–10322754	391	42.76	5.18	I, II, III, IV	A	6
*AcPG33*	CEY00_Acc19726	17	13702759–13706569	463	51.08	5.01	I, II, IV	E	6
*AcPG34*	CEY00_Acc20040	18	5124083–5129843	480	52.77	4.88	I, II, IV	E	6
*AcPG35*	CEY00_Acc20245	18	11600456–11604800	467	50.41	5.70	I, II, IV	E	4
*AcPG36*	CEY00_Acc21483	19	14524594–14527737	443	48.29	7.44	I, II, III, IV	A	6
*AcPG37*	CEY00_Acc22880	20	7747831–7753520	464	49.78	5.32	I, II, IV	E	7
*AcPG38*	CEY00_Acc22911	20	8264904–8267818	478	51.63	4.69	I, II, III, IV	A	6
*AcPG39*	CEY00_Acc23401	20	17590317–17598433	495	55.56	8.84	I, II, IV	E	5
*AcPG40*	CEY00_Acc24984	22	12041773–12043962	389	41.29	8.34	I, II, III, IV	C	4
*AcPG41*	CEY00_Acc24985	22	12053150–12055550	389	41.37	8.88	I, II, III, IV	C	4
*AcPG42*	CEY00_Acc24986	22	12063257–12065692	389	41.29	8.71	I, II, III, IV	C	4
*AcPG43*	CEY00_Acc25490	23	543417–548016	462	50.45	5.96	I, II, IV	E	6
*AcPG44*	CEY00_Acc22128	23	16932824–16938631	488	54.56	8.78	I, II, IV	E	5
*AcPG45*	CEY00_Acc26873	23	25969453–25974372	487	54.42	8.95	I, II, IV	E	5
*AcPG46*	CEY00_Acc31576	28	922828–928942	489	51.92	5.06	I, II, III, IV	D	7
*AcPG47*	CEY00_Acc32814	29	5177508–5182530	530	57.45	8.55	I, II, III, IV	A	7
*AcPG48*	CEY00_Acc33079	29	10363087–10368847	480	52.03	5.31	I, II, IV	E	7
*AcPG49*	CEY00_Acc33083	29	10413690–10417991	429	46.18	9.59	I, II, III, IV	D	5
*AcPG50*	CEY00_Acc33084	29	10418902–10421951	423	44.09	9.05	I, II, III, IV	D	5
*AcPG51*	CEY00_Acc33179	29	11728474–11731193	478	51.59	4.75	I, II, III, IV	A	6

## Supplementary Materials

The following are available online at https://www.mdpi.com/2223-7747/9/3/327/s1. **Figure S1**. PG family in chr map. **Table S1**. Motifs information identified by MEME from 51 PG proteins of kiwifruit. **Table S2**. Transcriptome FPKM results of 41 AcPG genes during kiwifruit ‘Donghong’ softening. **Table S3**. qPCR results of 26 AcPG genes during kiwifruit ‘Donghong’ softening. **Table S4**. Correlation analysis of gene expression and enzyme activity of AcPGs during ‘Donghong’ softening. **Table S5**. List of primers used for qPCR assay of AcPG genes.
