# Supplementary material for: Genome-Wide Identification and Expression Analysis of Polygalacturonase Gene Family in Kiwifruit (Actinidia chinensis) during Fruit Softening
Source: Plants (Basel). 2020 Mar 4;9(3):327. doi: 10.3390/plants9030327 (PMC7154832; doi:10.3390/plants9030327)
Supplement: Supplementary file 1 [file plants-09-00327-s001.pdf]

plants

MDPI

Supplementary Material

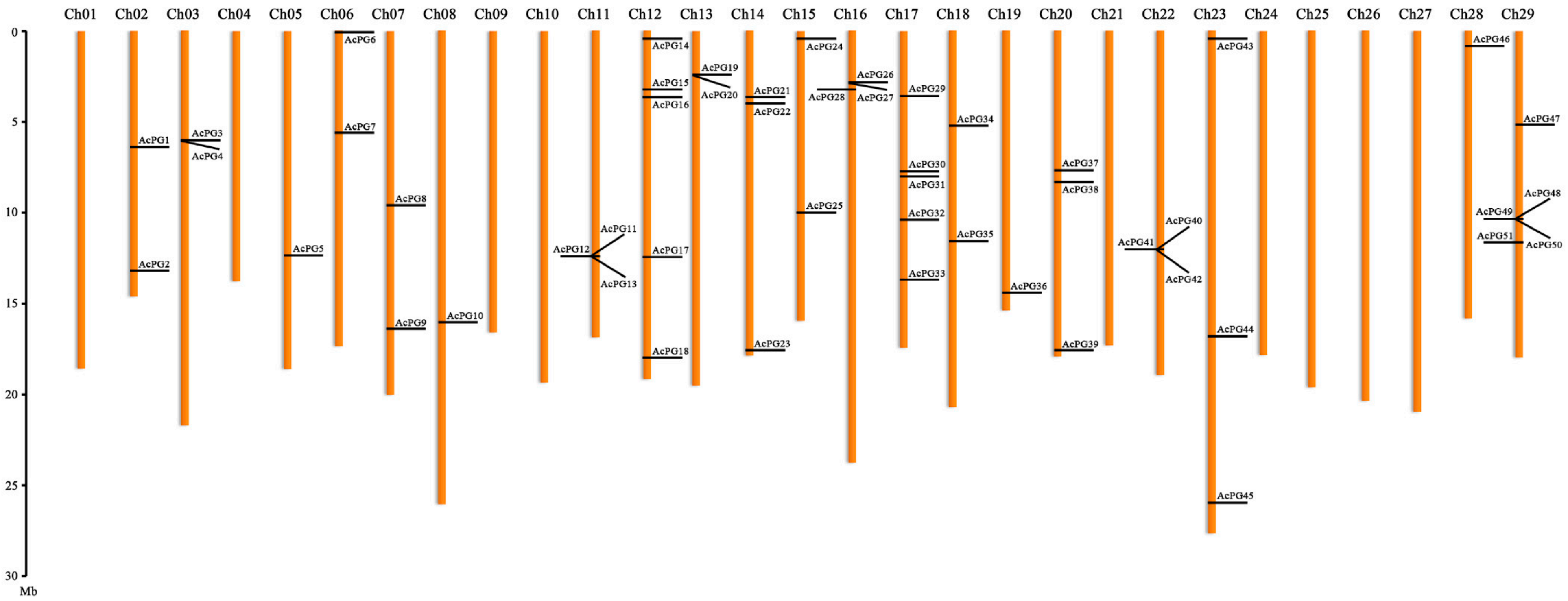

Figure S1. PG family in chr map.

Table S1. Motifs information identified by MEME from 51 PG proteins of kiwifruit.

Motifs	Width	Best Possible Match	Pfam Annotation	PG Domains
Motif1	41	APGDSPNTDGIHIDSSTNVVIEBSNIGTGDDCISIGSGWSN	GH28	I, II
Motif2	34	QGGGGYVRGITFQBITMENVKNPIIIDQNYCDHP	GH28	
Motif3	50	LTGSFNLTSHMTLFLARDAVILGSQDEKEWPLIEPLPSYGRGRELPGGRY	No hit	
Motif4	18	NITCGPGHGISIGSLGKS	GH28	III
Motif5	21	DFGAVGDGVTDNTKAFQSAWK	No hit	
Motif6	21	NVTVENVTLTNTTNGVRIKTW	GH28	IV
Motif7	15	GGGTIDGQGSKWWAK	No hit	
Motif8	41	KTRPTLLEFMNSSNIVVSGJTILNSPQFHIHFVGCKNVRVQ	GH28	

Table S2. Transcriptome FPKM results of 41 AcPG genes during kiwifruit 'Donghong' softening.

Gene_id	W0	W1	W2	W3	W4	W5	W6
AcPG1	0	0	0	0	0	0.005964	0.005943
AcPG2	0	0.012157	0.088802	0.076917	0.060583	0.026955	0.031771
AcPG3	0.005687	0	0	0	0	0	0
AcPG4	40.17887	68.95563	323.5688	568.4539	1060.107	1208.523	283.9159
AcPG6	0.082534	0.145185	0.145839	0.107986	0.098876	0.198089	0.242513
AcPG7	0	0.039278	0	0.004945	0.025012	0.009907	0.012505
AcPG8	0.201132	0.291321	0.726393	1.734398	1.705668	0.834505	1.090468
AcPG9	0	0	0.010639	0.004889	0.059254	0.047787	0.123419
AcPG10	0	0	0	0	0	0	0.003831
AcPG11	4.933503	3.779318	3.154088	2.145332	2.07919	3.373734	4.744524
AcPG12	0	0.009898	0	0.010018	0	0	0
AcPG13	0	0.004515	0	0	0.109432	0.038187	0
AcPG14	6.530088	4.199244	3.111557	5.219298	3.228238	2.248606	3.342678
AcPG15	9.598302	7.620523	6.530044	6.941791	5.605419	4.120339	7.487684
AcPG16	0	0	0	0	0.005174	0	0
AcPG18	45.41771	177.34	1694.221	2717.512	2848.047	4475.037	2251.134
AcPG19	0.024358	0.036823	0.150901	0.032678	0.02915	0.00727	0.00666
AcPG20	0	0.004839	0	0	0	0	0
AcPG21	2.808134	1.128272	0.218757	0.144839	0.239007	0.028834	0.024011
AcPG22	0.237985	0.172001	0.169042	0.047623	0.052688	0.05837	0.076554
AcPG24	12.10591	12.67585	8.925292	6.612047	7.782419	8.073493	12.41502

AcPG25	0	0	0	0	0.032461	0.01487	0.014486
AcPG26	0.473029	0.341556	0.037285	0.082194	0.097798	0.037354	0.097953
AcPG27	0.061149	0.008108	0.012899	0.004371	0.007578	0.00413	0.062608
AcPG28	1.805997	1.465551	1.05026	0.316238	0.499769	0.790778	1.370556
AcPG30	0.004953	0.005266	0	0	0	0	0
AcPG31	0.004896	0.009819	0.005126	0	0.022554	0	0.003748
AcPG32	0.005551	0.005902	0.022848	0.022381	0.009524	0.02115	0
AcPG33	1.46235	0.996372	0.617987	0.717085	0.896082	1.075926	0.618712
AcPG34	0.100727	0.097711	0.066296	0.03595	0.087073	0.007938	0.039488
AcPG35	0.004097	0	0.004215	0	0.052712	0.007853	0.033153
AcPG36	0.019187	0.003711	0.004053	0	0.028141	0.028292	0.029292
AcPG37	0.197905	0.143773	0.158067	0.118313	0.169947	0.004132	0.07585
AcPG39	2.081924	1.747334	2.286361	2.454927	1.491299	2.452891	3.275163
AcPG43	0.771587	0.582909	0.448327	0.35229	0.603861	0.483203	0.589446
AcPG44	5.206603	5.472286	6.843636	5.95603	6.162002	6.376221	7.000991
AcPG45	2.979494	2.546972	2.085045	1.895882	1.439254	1.885178	2.769173
AcPG47	0	0.003983	0.012699	0.028314	0.056203	0.065316	0.024207
AcPG48	1.387424	0.743037	0.163055	0.021072	0.648054	0.008883	0.052222
AcPG49	0	0	0	0.00929	0.003953	0.016503	0.004691
AcPG51	0.87742	0.842924	0.503413	0.089986	0.021981	0.024722	0.010626

Table S3. qPCR results of 26 AcPG genes during kiwifruit 'Donghong' softening.

Gene_id	W0	W1	W2	W3	W4	W5	W6
AcPG2	0.000	0.000	0.035	0.022	0.021	0.018	0.012
AcPG4	53.948	225.184	2005.531	2801.123	3137.239	4107.495	1677.472
AcPG6	0.021	0.015	0.012	0.016	0.010	0.018	0.025
AcPG8	0.123	0.204	0.469	1.136	1.177	0.533	0.488
AcPG9	0.000	0.000	0.007	0.013	0.025	0.026	0.018
AcPG11	1.521	1.098	0.797	0.513	0.297	0.595	1.563
AcPG13	0.027	0.000	0.004	0.000	0.117	0.003	0.006
AcPG14	0.442	0.271	0.251	0.384	0.219	0.155	0.196
AcPG15	0.828	0.572	0.523	0.616	0.447	0.319	0.541
AcPG18	12.687	93.116	1156.853	1245.736	823.492	1363.581	633.394
AcPG19	0.020	0.026	0.052	0.036	0.028	0.017	0.031
AcPG21	0.703	0.455	0.074	0.032	0.046	0.017	0.008

AcPG22	0.023	0.016	0.021	0.009	0.005	0.011	0.012
AcPG24	5.288	5.883	3.491	2.198	2.355	2.851	5.266
AcPG26	0.093	0.048	0.014	0.018	0.059	0.011	0.017
AcPG28	0.291	0.162	0.128	0.079	0.068	0.136	0.287
AcPG33	0.326	0.274	0.117	0.127	0.213	0.208	0.136
AcPG34	0.015	0.014	0.017	0.008	0.007	0.005	0.007
AcPG37	0.051	0.032	0.031	0.033	0.050	0.012	0.023
AcPG39	0.590	0.493	0.427	0.524	0.234	0.446	0.850
AcPG43	0.189	0.176	0.105	0.068	0.077	0.067	0.110
AcPG44	0.879	0.997	1.163	1.030	0.710	0.718	0.960
AcPG45	0.030	0.014	0.008	0.097	0.864	0.859	0.066
AcPG47	0.000	0.000	0.069	0.085	0.339	0.222	0.066
AcPG48	0.581	0.373	0.052	0.023	0.302	0.005	0.019
AcPG51	0.303	0.369	0.268	0.048	0.003	0.011	0.009

Note: values are mean from three replicates, and standard errors are not shown.

Table S4. Correlation analysis of gene expression and enzyme activity of AcPGs during 'Donghong' softening.

Gene Name	Pearson Coefficient	
	qPCR and FPKM	qPCR and PG Enzyme Activity
AcPG2	0.931	0.539
AcPG4	0.939	0.842
AcPG6	0.499	−0.591
AcPG8	0.975	0.810
AcPG9	0.658	0.847
AcPG11	0.959	−0.845
AcPG13	0.902	0.636
AcPG14	0.972	−0.579
AcPG15	0.961	−0.768
AcPG18	0.865	0.627
AcPG19	0.861	−0.002
AcPG21	0.973	−0.721
AcPG22	0.939	−0.851
AcPG24	0.990	−0.789
AcPG26	0.850	−0.303

AcPG28	0.869	−0.748
AcPG33	0.931	−0.350
AcPG34	0.714	−0.648
AcPG37	0.921	−0.081
AcPG39	0.835	−0.642
AcPG43	0.730	−0.811
AcPG44	0.180	−0.548
AcPG45	−0.714	0.848
AcPG47	0.911	0.958
AcPG48	0.994	−0.396
AcPG51	0.976	−0.722

Table S5. List of primers used for qPCR assay of AcPG genes.

Gene Name	Gene Locus	PCR Length	Sense Primer (5′-3′)	Anti-sense Primer (5′-3′)
AcPG2	CEY00_Acc02429.1	183	TCCTAGTCAAACCCGTCTCATTCTC	CCTGTCCATCAATAATACCGCTACC
AcPG4	CEY00_Acc03126.1	152	GCACGCTACGATGGTCAATGTC	CCGCAACTTCTGTCGCTGATG
AcPG6	CEY00_Acc06176.1	116	GTAGCACCAGATGAGCCGAATAAC	CTTCCACAGCCATCAATAACTCCAC
AcPG8	CEY00_Acc08039.1	115	GGATGTGGTGGGAGATGTGGTG	GGAGAGTTACAGAAGGTGAGGTTGG
AcPG9	CEY00_Acc08167.1	102	CCACGGTTTCTAAGGTCTTTGATACTC	GTGTGACTATTCCTGTGGAGTTGTTC
AcPG11	CEY00_Acc12552.1	156	CAATATTACTGTGATGTCAGAGGTGGATG	CAACAATATATCGGTGCAGGCAACAG
AcPG13	CEY00_Acc12562.1	82	TGGACGGACGAGGAAGAATTGATG	CGACATGGCTTACTTGTGTTGACC
AcPG14	CEY00_Acc12941.1	121	GTCTCGGCAATTTCCAAGCTGATG	CGCCTTCAGTTAGGAACAGAGTCATG
AcPG15	CEY00_Acc13137.1	125	AGAATGTGATGGTGAGTTGGAATCG	GGCTGGCTGAGTGAGGTTGAC
AcPG18	CEY00_Acc13940.1	176	GCAACCACTGACTTCTCTTCTTCTC	GCATAGCCGAACCGCAAGC
AcPG19	CEY00_Acc14204.1	101	CTACCATCACCACCTCCACTTCC	GGCGGGCTGTTGTCTTTCTTTG
AcPG21	CEY00_Acc15593.1	190	GGAGATGTGGAGGCGGAGAAC	CGTATTGGGAGAGTCGGTTGGAG
AcPG22	CEY00_Acc15651.1	188	AGGCCTTCAGGACGGCGATAAC	GGGATCTACCACAGGCCATTCGT
AcPG24	CEY00_Acc16389.1	161	GCCGATGTGGTAACTTGCTCTG	GTGCCGCCGCTACTCCTC
AcPG26	CEY00_Acc17817.1	132	AGGCAACTCTTCGGTTATCCAGATC	GACAGAATGGCTCCATCAACACTTATG
AcPG28	CEY00_Acc17848.1	123	GCCCACCACCATCAGCTTTATG	TCGACACTAAGCCCATCCACATCC
AcPG33	CEY00_Acc19726.1	125	CCAGGTTGCTGTCCAGAATGTG	GGTCGTCTCCAGTGCTAATGTAAC
AcPG34	CEY00_Acc20040.1	93	GCCGAGGAAGGGACACGACTG	TCGTGCCGTTGTCGCCTGTTAC
AcPG37	CEY00_Acc22880.1	182	CCAGATTCGTCGGTTAGTGTATGC	TCACTGCCAAGAGCGAGAGC
AcPG39	CEY00_Acc23401.1	179	ACAGGAACCTAACCTTCAACAATGTC	GATTTCTACGCACCCGTGGATC
AcPG43	CEY00_Acc25490.1	106	CCGCTCGTGAAGAGGCTAGTG	AAGTCCTACTGTATTCGCATTGTCC

AcPG44	CEY00_Acc22128.1	180	GGGATTCGTGGTCATGGTGTTCG	CTCACACGGAGTTGGGAAAATCC
AcPG45	CEY00_Acc26873.1	167	CAATGATCCTCTCATCGCACCAAAC	CAGATTGAATGCCACCGATCTAAGC
AcPG47	CEY00_Acc32814.1	99	CCATCACCACCACCTCTAAAAGC	GGAGGGTGGTTTGTTGTCTTTC
AcPG48	CEY00_Acc33079.1	165	GGATTGCCTATGACAGACCTACCG	GGAACTCAACACCGCAAAATGAG
AcPG51	CEY00_Acc33179.1	180	ATGTGACAGCCCTGCCCTAATTC	TCGATGTGAATCCCATCGGTATTG
AcActin	CEY00_Acc08081.1	120	GCAGGAATCCATGAGACTACC	GTCTGCTATACCAGGGAACAT
